# *Erythema migrans*-like lesions associated with *Borrelia afzelii* infection in a European badger, *Meles meles*

**DOI:** 10.3389/fvets.2025.1636700

**Published:** 2025-09-18

**Authors:** Andrei Daniel Mihalca, Georgiana Deak, Cristina Daniela Cazan, Noureddine Mechouk, Andrada Negoescu, Marian Taulescu, Călin Mircea Gherman

**Affiliations:** ^1^Department of Parasitology and Parasitic Diseases, University of Agricultural Sciences and Veterinary Medicine of Cluj-Napoca, Cluj-Napoca, Romania; ^2^Department of Pathology, University of Agricultural Sciences and Veterinary Medicine of Cluj-Napoca, Cluj-Napoca, Romania

**Keywords:** European badger, *Erythema migrans*, Lyme borreliosis, ticks, wildlife disease

## Abstract

**Introduction:**

*Erythema migrans* (EM), a characteristic skin lesion, is a well-known clinical outcome of Lyme borreliosis in humans, caused by various *Borrelia burgdorferi* sensu lato (s.l.) species. However, its occurrence in animals, mainly wildlife, has been largely unexplored. This report presents a possible case of EM in a European badger (*Meles meles*) from Romania.

**Materials and methods:**

The badger exhibited multiple erythematous lesions on its ventral body surface, and these lesions were heavily infested with *Ixodes ricinus* ticks. Histological examination of skin biopsies was conducted to assess inflammatory reactions. Molecular analysis was performed to detect *Borrelia* DNA in the lesions.

**Results:**

Histological examination revealed a mild, chronic inflammatory reaction consistent with EM-like lesions as observed in humans. Molecular analysis confirmed the presence of *Borrelia afzelii* DNA in the skin lesions.

**Conclusion:**

These findings underscore the importance of wildlife surveillance in understanding the ecoepidemiology and pathogenesis of Lyme borreliosis. Badgers, as potential reservoir hosts, may play a role in the disease cycle.

## 1 Background

*Erythema migrans* (EM) is the most common sign among the several clinical outcomes of Lyme borreliosis (LB) in humans and has been defined in various ways by different authorities (1). According to Stanek et al. (2), in Europe, EM is defined as an “expanding red or bluish-red patch (≥5 cm in diameter), with or without central clearing” that develops days to weeks after the bite of a vector *Ixodes* spp. tick infected with *Borrelia burgdorferi sensu lato* (s.l.) (1). If two or more erythematous skin lesions are present in association with a tick bite, to fulfill the clinical definition for multiple EM, one of these should be at least 5 cm in diameter (1). However, for smaller lesions to be defined as EM, they should be associated with a tick bite history and a delay in appearance of at least 2 days (1).

In animals, clinical Lyme borreliosis (LB) is known in dogs and horses, with occasional reports in domestic ruminants and cats, but most seropositive cases remain asymptomatic (3). In dogs, the most common clinical manifestations include fever, lameness associated with arthritis, general malaise, or renal failure (17). In horses, clinical LB includes signs such as malaise, fever, stiffness, laminitis, arthritis, and uveitis (3). However, skin lesions have been rarely reported in association with LB in animals, and there is no clinical case definition for EM in veterinary medicine. Dogs infected with *B. burgdorferi* develop a small reddish lesion at the site of tick attachment, which disappears within 1 week (17). In addition, a report mentions two dairy cows infected with *Borrelia afzelii* and *B. burgdorferi* sensu stricto (s.s.), respectively, which developed erythema on the skin of the udder (4).

Although wild animals are commonly infected with ticks, pathology associated with the infection with *B. burgdorferi* sensu lato (s.l.) has not been reported. The aim of this study is to report *Erythema migrans-like* lesions in a European badger infected with *Borrelia burgdorferi* sensu lato.

## 2 Materials and methods

### 2.1 Sample collection and tick identification

An adult female European badger, *Meles meles*, was collected by legal hunting on 30 October 2021 in Adea, Arad County, Romania (46.57N, 21.63E) and transported under refrigeration to the Department of Parasitology and Parasitic Diseases. A full postmortem parasitological necropsy was performed, as part of a larger survey of wild carnivores from Romania. All ectoparasites, including ticks, were collected and fixed in 70% ethanol for further identification. All ticks were identified according to morphological keys by Estrada-Peña et al. (5). Two skin tissue samples with erythematous lesions were also collected using a scalpel blade in 100% ethanol and 10% buffered formalin, respectively. The formalin-preserved skin samples were paraffin-embedded. Serial 2-μm sections were cut and routinely processed for hematoxylin and eosin (HE) staining.

### 2.2 DNA extraction and molecular identification

DNA was isolated from a 2-mm skin biopsy sample using the ISOLATE II Genomic DNA Kit (Bioline Meridian Bioscience, Luckenwalde, Germany), according to the manufacturer’s instructions, and stored at −20 °C. The extract was screened by nested polymerase chain reaction (PCR) targeting a portion of the conserved 41-kDa chromosomal flagellin gene (flaB) from *B. burgdorferi* s.l. The first PCR amplification of the flagellin (flaB) gene region (~650 bp) of *B. burgdorferi* s. l. was performed in a 25-μl reaction volume, containing 12.5 μL of Green PCR MasterMix (Rovalab GmbH, Teltow, Germany), 6.5 μL of ultrapure water, 1 μL (10 pmol/μL) of each of the two primers FlaLL (5′-ACATATTCAGATGCAGACAGAGGT-3′) and FlaLS (5′-AACAGCTGAAGAGCTTGGAATG-3′) (6), and 4 μL aliquot of isolated DNA. One negative control (ultra-pure water) was included. The PCR was performed using the T1000™ Thermal Cycler (Bio-Rad, London, UK) with the following conditions: initial denaturation at 95 °C for 5 min, followed by 35 cycles of denaturation at 95 °C for 30 s, annealing at 55 °C for 30 s, and extension at 72 °C for 45 s, with a final extension at 72 °C for 5 min. The PCR amplification was followed by a nested PCR targeting the same gene region (~350 bp) performed in a 25-μl reaction volume, containing 12.5 μL of Green PCR MasterMix (Rovalab GmbH, Teltow, Germany), 8.5 μL of ultrapure water, 1 μL (10 pmol/μL) of each of the two primers FlaRS (3′-CGATAATCTTACTATTCACTAGTTTC-5′) and FlaRL (3′-TGTTAGACGTTACCGATACTAACG-5′), and 2 μL of the first PCR reaction mix. A second negative control (ultra-pure water) was included. The nested PCR was performed with the following conditions: initial denaturation at 95 °C for 5 min, followed by 35 cycles of denaturation at 95 °C for 30 s, annealing at 59 °C for 30 s, and extension at 72 °C for 30 s, with a final extension at 72 °C for 5 min. Amplification products were visualized by electrophoresis on a 1.5% agarose gel stained with ECO Safe 20,000 × Nucleic Acid Staining Solution (Pacific Image Electronics, New Taipei, Taiwan), and their molecular weight was assessed by comparison with a molecular marker (O’GeneRuler™ 100 bp DNA Ladder, Thermo Fisher Scientific Inc., Waltham, MA, USA). The PCR product was purified using the ISOLATE II PCR and Gel Kit (Bioline Meridian Bioscience, Luckenwalde, Germany) and sent for sequencing (Macrogen Europe, Amsterdam, The Netherlands). The sequence was compared with those available in GenBank™ using Basic Local Alignment Tool (BLAST) analyses. The sequence was analyzed and edited using Geneious® 4.85 software (7).

## 3 Results

**Figure 1:**
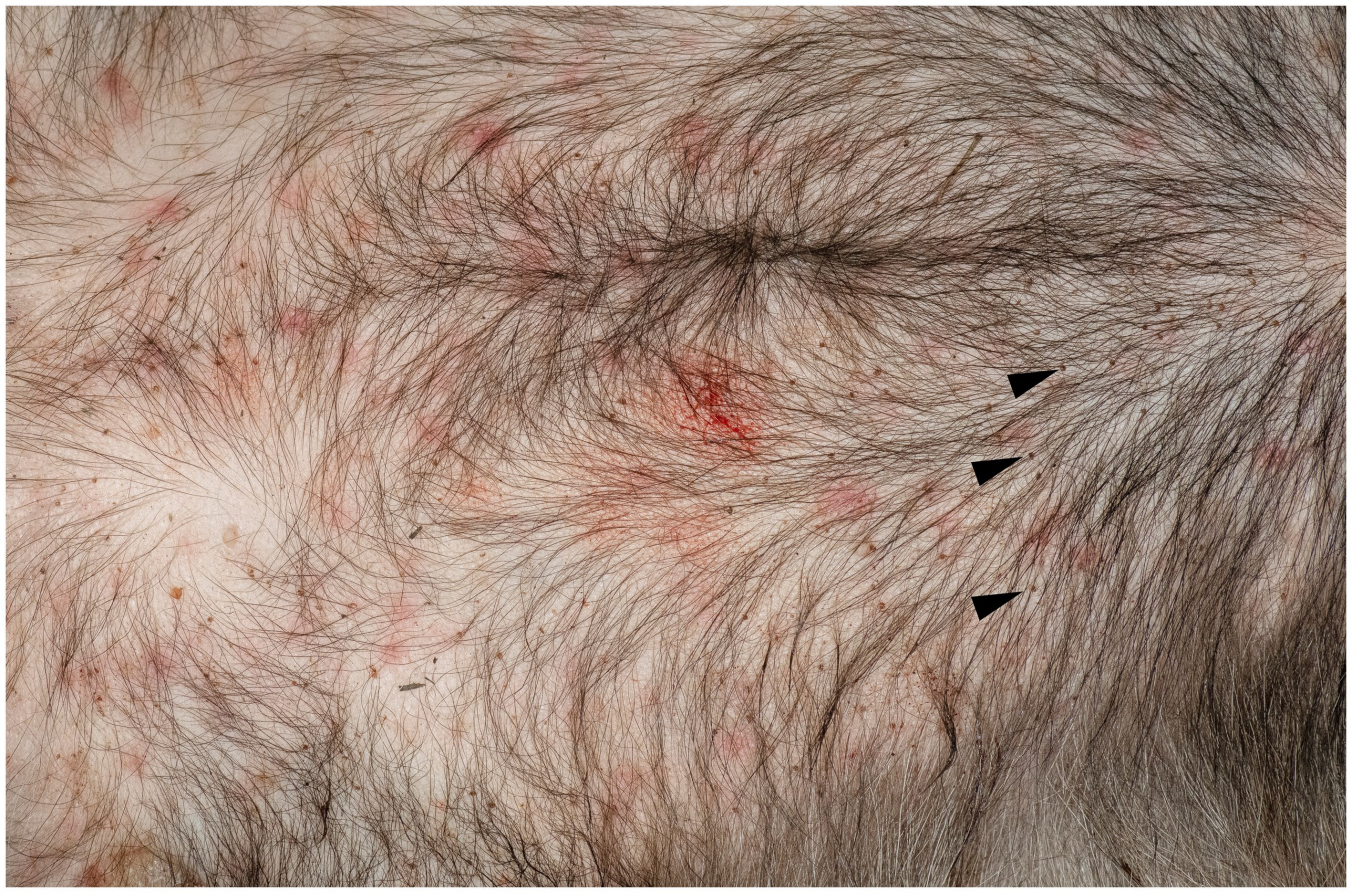
Erythematous skin lesions in the ventral body are of a European badger, *Meles meles*. Note also the presence of a large number of nymphs of *Ixodes ricinus* (arrow head).

**Figure 2:**
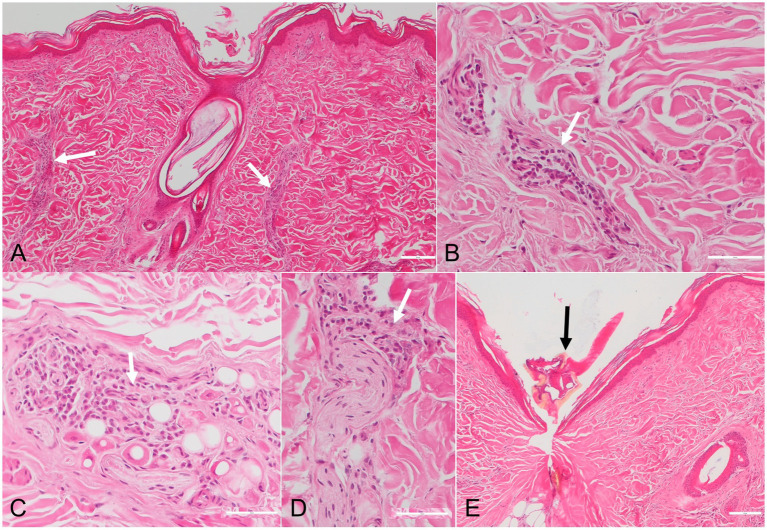
Photomicrographs of skin sections with chronic *Erythema migrans* in a European badger (*Meles meles*). The inflammatory infiltrates, consisting of small lymphocytes, plasma cells and rare eosinophils (white arrows), are present in the perivascular **(A,B)**, interstitial **(C)** and perineural **(D)** areas. Fragments of a tick are observed in one skin sample (black arrow) **(E)**. HE stain.

Upon the visual inspection of the skin of the badger, multiple erythematous lesions with variable irregular shapes and sizes, lacking central clearing, were found on the ventral part of the body (Figure 1). The area was heavily infested with ticks. Overall, 211 ticks were removed, and all were morphologically identified as *Ixodes ricinus* (7 larvae, 199 nymphs, and 5 females). Histological examination of the skin biopsies revealed the presence of a mild, multifocal (Figure 2A), chronic inflammatory reaction in both the superficial and deep dermis, predominantly perivascular (Figures 2A,B), interstitial (Figure 2C), and perineural (Figure 2D). The inflammatory cells were represented by small lymphocytes and plasma cells with rare eosinophils. The overlying epidermis was intact. In one biopsy, small fragments of a tick were observed (Figure 2E).

The DNA sample of the evaluated skin biopsy tested positive for *B. burgdorferi* sensu lato. The positive sample was successfully sequenced and phylogenetically analyzed for the flagellin (flaB) targeted gene. The sample clustered within *B. afzelii* and showed 100% identity with all reference *B. afzelii* isolates from Poland (KY626325, MG944962, MK604269, MK604271), Italy (KY213885), Spain (KT347442), Portugal (KJ810661), Turkey (MK922618, MK922620), and Russia (MT007936, MT007938, MT007941).

## 4 Discussion

This study provides the first report of EM-like lesions in wildlife. In humans from the United States, EM has been associated with *B. burgdorferi* sensu stricto. However, in Europe, human EM is mostly caused by *B. afzelii* and *Borrelia garinii*, more rarely by *B. burgdorferi* s.s., and exceptionally by *Borrelia bissettii* and *Borrelia spielmanii* (1). Similarly, in the present report, the EM-like lesions of the badger were associated with the presence of *B. afzelii* DNA. *Borrelia burgdorferi* s.l. was reported in European badgers only on very few occasions. A study from Switzerland identified *B. afzelii* and *Borrelia valaisiana* by PCR/restriction fragment length polymorphism (RFLP) in the skin of six out of eight examined European badgers. Moreover, live spirochetes were isolated in cultures, suggesting that the two *Borrelia* species are able to produce an active infection in this host (8). In Poland, 12% of the examined European badgers were infected with *Borrelia burgdorferi* s.l., with only *B. afzelii* being detected (9). *B. burgdorferi* s.l. DNA was also detected in the tissues of 0.9% of European badgers in the Netherlands (10). Despite these reports (including the isolation of live Lyme spirochetes and our record of EM), the role of badgers as reservoir hosts has not been confirmed by xenodiagnoses (8).

European badgers are hosts to various tick species, some of which have been demonstrated as suitable vectors for *B. burgdorferi* s.l. *I. ricinus*, the main vector of *B. burgdorferi* sensu lato. In Europe, badgers have been reported on various occasions in Poland (9), Switzerland (8), Spain (11), the Netherlands (10), and Romania (12). European badgers are also common hosts of another Lyme *Borrelia* vector, *Ixodes hexagonus,* with reports from various countries in Europe (9, 10, 12).

The pathogenesis of EM in humans is related to the cutaneous inflammation caused by the centrifugal dissemination of the spirochetes from the site of the tick bite, while the more distant, secondary EM lesions are associated with their hematogenous spread to other skin locations (1). The lesions seem to be related to a strong cell-mediated immune response (1). Our histological findings align with those of chronic EM associated with LB, as reported in humans. However, since no clinical evaluation was performed to monitor the lesions over several days, we classify them as potential EM. As such, EM is characterized by a perivascular lymphocytic inflammatory infiltrate present in both the superficial and deep dermis, with plasma cells present at the periphery of the inflammation and eosinophils in the center (13). The presence of plasma cells throughout the dermis is more specific to chronic EM (14). Furthermore, eosinophils are more common in early lesions, compared to plasma cells, which are more frequent in late lesions (15).

## 5 Conclusion

While EM is well-documented in humans, this is the first suspected case reported in wildlife, potentially expanding our understanding of the disease’s distribution and presentation in nature. This report also provides new circumstantial evidence, in addition to other data, such as the common presence of suitable vector ticks, that badgers could play a role in the epidemiology of LB.

## Data Availability

All presented data is available on reasonable request from the corresponding author.
